# Obesity and Cancer Metabolism: A Perspective on Interacting Tumor–Intrinsic and Extrinsic Factors

**DOI:** 10.3389/fonc.2017.00216

**Published:** 2017-09-14

**Authors:** Steven S. Doerstling, Ciara H. O’Flanagan, Stephen D. Hursting

**Affiliations:** ^1^Department of Nutrition, University of North Carolina at Chapel Hill, Chapel Hill, NC, United States; ^2^Lineberger Comprehensive Cancer Center, University of North Carolina at Chapel Hill, Chapel Hill, NC, United States; ^3^University of North Carolina Nutrition Research Institute, Kannapolis, NC, United States

**Keywords:** obesity, cancer, cancer metabolism, growth factors, inflammation, autophagy, calorie restriction

## Abstract

Obesity is associated with increased risk and poor prognosis of many types of cancers. Several obesity-related host factors involved in systemic metabolism can influence tumor initiation, progression, and/or response to therapy, and these have been implicated as key contributors to the complex effects of obesity on cancer incidence and outcomes. Such host factors include systemic metabolic regulators including insulin, insulin-like growth factor 1, adipokines, inflammation-related molecules, and steroid hormones, as well as the cellular and structural components of the tumor microenvironment, particularly adipose tissue. These secreted and structural host factors are extrinsic to, and interact with, the intrinsic metabolic characteristics of cancer cells to influence their growth and spread. This review will focus on the interplay of these tumor cell–intrinsic and extrinsic factors in the context of energy balance, with the objective of identifying new intervention targets for preventing obesity-associated cancer.

## Introduction

Cancer is a disease characterized by dysregulated cell growth, whereby critical genetic mutations unleash limitless replicative potential (1). These genetic alterations drive tumor growth by engaging various signaling pathways involved in cell proliferation, angiogenesis, inflammation, invasion, and evasion of apoptosis (2). However, sustained cell proliferation requires availability of adequate energy and molecular building blocks for generating daughter cells. Therefore, oncogenic transformation is commonly accompanied by simultaneous metabolic reprogramming of the cell’s carbon economy to support cell growth (3–5). Accordingly, substantial scientific effort has been marshaled to deconvolute the complexities of cancer metabolism.

A robust body of evidence demonstrates that many cancer cells, in contrast to normal cells, subsist primarily (although not exclusively) on glucose metabolism *via* aerobic glycolysis, regardless of oxygen availability (5, 6). This phenomenon, termed the Warburg effect, involves restricting the majority of carbohydrate substrate to glycolysis, rather than the efficient ATP generation *via* oxidative phosphorylation. In this way, the carbon skeletons of glucose can be invested in a variety of biosynthetic pathways necessary for daughter cell production. In fact, this tradeoff—catabolic efficiency for anabolic utility—is a characteristic of eukaryotic cells undergoing proliferation, such as activated T cells (7). Furthermore, cancer cells exhibit compensatory increases in glucose uptake to account for this compromise (6). Common to many oncogene-driven signaling pathways is the activation of transcriptional programs that upregulate enzymes of glycolysis, the pentose phosphate pathway (PPP), fatty acid biosynthesis, and protein synthesis (8). Metabolic priorities are thus aligned to meet the various demands of cell proliferation, including nucleotide synthesis for DNA replication, fatty acid biosynthesis for cell membrane expansion, and protein synthesis for duplication of the intracellular proteome. Therefore, metabolism has emerged as a critical mediator between genomic alterations and the metabolic orchestration of complex cellular growth patterns intrinsic to the cancer cell.

In addition to internally restructuring metabolic activity, cancer cells encounter a variety of extrinsic factors that can support or suppress tumorigenesis (3, 9). Obesity is attendant to profound metabolic changes that promote tumor growth. High serum levels of insulin and insulin-like growth factor 1 (IGF-1), adipose tissue dysfunction and inflammation, and nutrient-replete circulation constitute several of the mechanisms by which obesity supports malignant cell growth (9, 10). Obesity-associated systemic signals serve dual roles for cancer metabolism. In one respect, these factors “fuel the fire” of oncogene-induced metabolic reprogramming by supplying ample substrate and supportive growth-factor signaling. On the other hand, this deluge of obesity-associated signals contributes to the restructuring of enzymatic networks that drive cancer metabolism intrinsic to the cell. To address this relationship, calorie restriction (CR), commonly defined as 10–40% reduction in calorie intake without nutrient deficiencies, has shown auspicious results in numerous studies gauging cancer protective potential (11–13). By means of attenuating obesity-associated signaling pathways and activating nutrient stress responses, CR regimens present a unique lifestyle approach to reduce obesity-associated cancer risk. Indeed, efforts to imitate the mechanisms of CR have given rise to a class of nutrients and pharmacologic agents collectively known as CR mimetics (13). In light of evidence that cancer cells respond dynamically to host metabolism, it is necessary to recognize these extrinsic signals as a frontier for novel preventive and therapeutic paradigms.

The objective of this narrative review is to summarize landmark developments in our understanding of cancer metabolism relevant to the mechanisms underlying the impact of obesity on cancer. We will also consider the emerging role of diet to complement traditional pharmacologic therapies directed against cancer metabolism. The fundamental challenge in targeting cancer cell metabolism is the inability of therapeutic agents to discriminate their effects between normal and cancerous cells. This occurs because normal tissues often share aspects of metabolic activity with tumors, resulting in adverse side effects. Conversely, dietary approaches are well-tolerated by the body and pose few deleterious side effects. Therefore, it is critical to devise combinatorial treatment regimens that simultaneously leverage the safety of dietary approaches and the specificity of pharmacologic agents. Lastly, recent work has drawn attention to the complementary extrinsic and intrinsic features in cancer metabolism (3, 9). Here, we apply a similar framework to identify points of convergent signaling, and thus targetable vulnerabilities, in the obesity–cancer link.

## Growth Factors and Their Signals

### Insulin, IGF-1, and Cancer: Epidemiologic Evidence

A series of complex hormonal signals superintend the distribution of energy in the body. Glucose is the body’s principal energy source, and glucose homeostasis is regulated largely by the peptide hormone insulin, which is secreted by pancreatic β-cells in response to hyperglycemia. Chronic obesity often results in insulin resistance, whereby insulin-responsive tissues fail to execute insulin signaling, prompting further insulin secretion (14). Accordingly, hyperinsulinemia is characteristic of the obese state (15).

Insulin also stimulates hepatic synthesis of the peptide IGF-1 (10). Synthesis of IGF-1 is likewise induced by growth hormone (16) and high protein diets (17, 18). Overweight individuals typically display increased circulating levels of IGF-1 (19), yet there are mixed associations with obesity (20). Importantly, IGF-1 activity is strongly regulated by IGF-binding proteins (IGFBPs), which bind upwards of 90% of all IGF-1 in circulation. These proteins restrict bioavailability by binding IGF-1 and precluding interactions with IGF-1 receptor (IGF-1R). Interestingly, IGFBP1 and IGFBP2 are regulated by nutrient-sensitive signals. Insulin suppresses IGFBP1 and IGFB2 (21–23), and serum levels of both factors are inversely related to BMI (19). Insulin-mediated suppression of IGFBP1 and IGFBP2 relieves their inhibitory effect on IGF-1, suggesting one possible mechanism by which hyperinsulinemia potentiates IGF-1 biologic activity. Of note, there is also mounting evidence for diverse IGF-1R-independent functions of IGFBPs with relevance to cancer (23).

Hyperinsulinemia is strongly associated with increased risk and progression in a variety of cancers, particularly those of the pancreas, endometrium, breast (postmenopausal), and colon (24). For instance, in a small prospective cohort of women with early-stage breast cancer, women in the highest versus lowest quartile of fasting insulin had adjusted hazard ratios of 2.1 (95% CI, 1.2 to 3.6) for disease recurrence and 3.3 (95% CI, 1.5 to 7.0) for mortality (25). Moreover, one prospective cohort study found that even among non-obese individuals, hyperinsulinemia was associated with a significant 89% increase in cancer mortality compared with non-obese individuals without hyperinsulinemia (26). This suggests a capacity of insulin signaling to guide tumor outcomes independent of body weight. Similar to insulin, epidemiologic studies largely demonstrate positive associations between serum IGF-1 and cancer risk and mortality, principally among colon and postmenopausal breast cancers (24, 27–30). Despite mechanistic implications for IGF-1 bioavailability, population-based studies have failed to capture a consensus on the impact of IGFBPs on cancer risk and outcomes (23).

### Regulation of Intrinsic Metabolic Pathways by External Growth Factors

Circulating insulin and IGF-1 primarily converge with intrinsic cancer cell metabolic processes *via* binding to their cell surface receptors, specifically insulin receptor (IR) and IGF-1R. Insulin can also bind and activate IGF-1R, and heterodimers of IR and IGF-1R can form (16). Both receptors possess tyrosine kinase activity and activate the canonical phosphatidylinositol 3-kinase (PI3K) signaling pathway (8, 16). Importantly, this pathway activates two transcription factors, hypoxia-inducible factor 1-alpha (HIF-1α) and Myc (31–34), that strongly upregulate metabolic processes conducive to cell proliferation. Both HIF-1α and Myc promote expression of enzymes in glycolysis (35–38). PI3K-mediated activation of Akt also acts to increase expression of glucose transporters and activate the kinase domain 6-phosphofructo-2-kinase/fructose-2,6-bisphosphatase 3 (PFKFB3), which catalyzes the formation of fructose-2,6-bisphosphate, allosteric activator of the key glycolytic enzyme phosphofructokinase-1 (39, 40). Accordingly, external activation of PI3K signaling by circulating growth factors can strongly promote a glycolytic phenotype.

**Figure 1:**
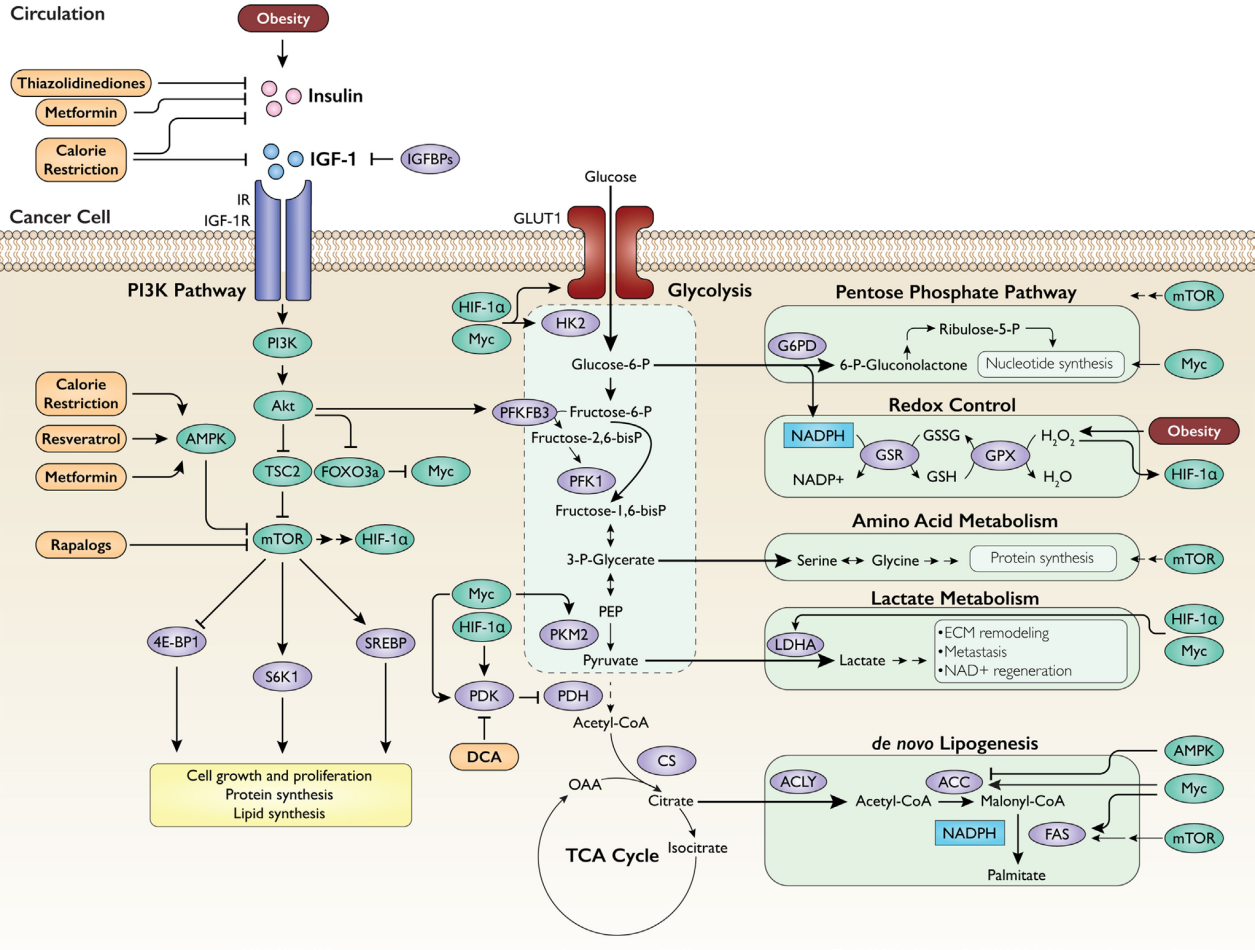
Regulation of intrinsic cancer metabolism by obesity-associated growth factors. Insulin and insulin-like growth factor 1 (IGF-1) activate the phosphatidylinositol 3-kinase (PI3K) pathway, which in turn upregulates glycolysis and subsidiary metabolic pathways to generate energy and fulfill the biosynthetic needs of proliferation. IGFBPs, IGF-binding proteins; IR, insulin receptor; IGF-1R, IGF-1 receptor; AMPK, AMP-activated protein kinase; TSC2, tuberous sclerosis 2; FOXO3a, forkhead box O3a; Myc, c-Myc; mTOR, mammalian target of rapamycin; HIF-1α, hypoxia-inducible factor 1-alpha; 4E-BP1, eukaryotic translation initiation factor 4E-binding protein 1; S6K1, ribosomal protein S6 kinase beta-1; SREBP, sterol regulatory element-binding protein; HK2, hexokinase 2; PFKFB3, 6-phosphofructo-2-kinase/fructose-2,6-bisphosphatase 3; PFK1, phosphofructokinase-1; PEP, phosphoenolpyruvate; PKM2, pyruvate kinase M2; PDK, pyruvate dehydrogenase kinase; PDH, pyruvate dehydrogenase; CS, citrate synthase; OAA, oxaloacetate; G6PD, glucose-6-phosphate dehydrogenase; GSR, glutathione reductase; GSX, glutathione peroxidase; LDHA, lactate dehydrogenase A; ECM, extracellular matrix; ACLY, ATP citrate lyase; ACC, acetyl-CoA carboxylase; FAS, fatty acid synthase; DCA, dichloroacetate. Intervention approaches are shown in orange.

Growth factor signaling also activates pathways that utilize intermediates from glycolysis to fulfill various biosynthetic needs of neoplastic growth. Glucose-6-phosphate is the first metabolite produced by glycolysis, and it serves as a substrate for the PPP to generate NADPH and nucleotides. NADPH plays a central role in carcinogenesis by fueling antioxidant systems, such as glutathione, allowing the cell to evade apoptosis (41). Nucleotide synthesis is necessary for DNA replication, the demand for which is heightened during the rapid production of daughter cells in cancer. Myc activates enzymes involved in nucleotide synthesis (42, 43), and the mammalian target of rapamycin (mTOR), a master regulator of cell growth downstream of PI3K/Akt, also activates the PPP (44). Moreover, Myc promotes the expression of the pyruvate kinase M2 isoform by alternative splicing (45), which allows upstream glycolytic intermediates to accumulate and feed into the production of biomass. A similar effect is also achieved by HIF-1α and Myc-mediated stimulation of pyruvate dehydrogenase kinase (PDK) (35, 38), which phosphorylates and inhibits pyruvate dehydrogenase (PDH), thereby redirecting pyruvate away from the tricarboxylic acid (TCA) cycle. Additional pathways that utilize glycolysis to support cell proliferation include: (a) amino acid synthesis from the glycolytic intermediate 3-phosphoglycerate stimulated by mTOR (46); (b) lactate synthesis through activation of lactate dehydrogenase A by HIF-1α and Myc (35, 37, 38); and (c) *de novo* lipogenesis supported by activation of acetyl-CoA carboxylase (ACC) by Myc (36, 47) and activation of fatty acid synthase (FAS) by Myc (48) and mTOR (49). An integrated schematic of these metabolic pathways is displayed in Figure 1.

### Targeting Growth Factor Signaling and Cancer Metabolism: Pharmacological and Dietary Examples

Given that growth factors can strongly affect the metabolic tone of cancer cells, therapies that address these signals from extrinsic and intrinsic perspectives hold promise to improve cancer prognosis, particularly for obese individuals. One approach entails reducing the abundance or activity of host growth factors, which would result in diminished activation of the corresponding intracellular signals. Metformin and thiazolidinediones (TZDs) are commonly prescribed to restore insulin sensitivity in type II diabetes, and patients taking either medication often experience improved response to chemotherapy (50–52) and lower cancer incidence (53, 54) in randomized trials. Moreover, metformin is a known activator of AMP-activated protein kinase (AMPK), and TZDs are ligands of peroxisome proliferator-activated receptor gamma. Therefore, it is difficult to isolate the tumor suppressive impact of these agents attributable to direct effects on metabolic signals in cells versus indirect effects on systemic factors such as reductions in circulating insulin.

Several dietary approaches can also decrease systemic growth factor levels and inhibit cancer development and progression. The best studied of these is CR, defined as a reduction of dietary energy intake without malnutrition (55–57). We and others have shown potent anticancer effects in various rodent models of cancer, and have linked the cancer preventive effects of CR to reduced IR and/or IGF-1 receptor signaling. However, despite a host of preclinical studies demonstrating the anticancer effects of CR, there is a lack of randomized trials investigating the impact of chronic CR on cancer risk and outcomes in humans, due in large part to the challenge in sustaining a chronic energy restricted diet. Recently, variations on CR, including intermittent CR regimens such as a 5:2 diet (5 days of a healthy diet and 2 days of a very low calorie/low carbohydrate 2 days/week), intermittent fasting, and ketogenic diets, have been tested in rodent models, each with some metabolic and anticancer effects similar to CR. Hopefully, some of these emerging approaches to dietary energy restriction will be more easily translated from animal to human studies.

An alternative strategy under evaluation is to use pharmacogical agents as so-called CR mimetics to induce the metabolic and anticancer effects of CR without the necessity of maintaining a restricted diet. For example, our group has demonstrated that chronically obese mice that underwent modest dietary weight loss demonstrated obesity-associated metabolic and inflammatory perturbations that were not fully reversed with the weight loss. However, treatment with the mTOR inhibitor Afinitor^®^ achieved favorable reductions in mTOR pathway signaling and mammary tumor growth compared with mice who only lost weight without Afinitor treatment (58). Similar approaches using metformin, which also inhibits mTOR pathway signaling as discussed above, are also being tested in preclinical and clinical studies to favorably reprogram metabolism and prevent cancer. Indeed, pharmacologic inhibition of PI3K/mTOR in an *in vitro* model of lung adenocarcinoma dramatically suppressed lactate production, glucose consumption, and PPP metabolites (59). In addition, administration of dichloroacetate, which inhibits PDK and thus reactivates PDH, has shown therapeutic potential in preclinical models and is being investigated in clinical studies (9, 60). Further research is needed on CR mimetics, particularly combinatorial regimens directed against multiple growth factor signaling and inflammatory pathways that are dysregulated with obesity.

One potential limit to the utility of merely reducing growth factor abundance is the frequency of genetic mutations in the PI3K pathway downstream of these signals. Somatic mutations in the *PIK3CA* gene, which encodes PI3K, are cell–intrinsic alterations in many cancers and often confer constitutive activation of the downstream mTOR signaling pathway (61). Preclinical studies of tumors with constitutively active PI3K (due to PIK3CA mutations) or mTOR (*via* genetic engineering) signaling were resistant to the anticancer effects of CR (62, 63). This highlights the importance of considering each tumor’s intrinsic genomic profile to identify appropriate candidates for growth factor reduction strategies.

## Adipose Tissue Dysfunction and Inflammation

### Epidemiologic Trends of Obesity, Chronic Inflammation, and Cancer

Chronic positive energy balance precedes the development of obesity, and excess energy is converted to triacylglycerol (TAG) and stored in adipose tissue. In consequence, adipocytes can become engorged with TAG, resulting in widespread metabolic dysfunction in obese individuals. The endocrine functions of adipose tissue are strongly impacted by this adipose tissue expansion. Leptin is a peptide hormone secreted primarily by adipose tissue in proportion to energy stores (64) and promotes energy homeostasis in part by activating satiety cues in the hypothalamus (65). Consequently, obesity is often accompanied by high circulating levels of leptin. Epidemiologic studies have demonstrated associations between high circulating levels of leptin and increased cancer risk, particularly for colon (66) and breast (67, 68) cancers. Adiponectin is another peptide hormone secreted by adipose tissue, but it is secreted in inverse proportion adiposity. Therefore, adiponectin levels are characteristically low in obese individuals (69). Lower circulating levels of adiponectin are associated with heightened risk of a variety of cancers (70), including colon (71) and postmenopausal breast (72, 73) cancers.

Adipose tissue is also the site of profound inflammatory activity. Adipose tissue hosts a characteristically high abundance of macrophages and other immune cells in obese versus lean subjects (74, 75). Immune cells in the stromal-vascular fraction of adipose tissue complement native expression of proinflammatory cytokines by adipocytes. In consequence, circulating levels of cytokines including tumor necrosis factor alpha (TNFα), interleukin-6 (IL-6), and IL-1β are higher in overweight and obese individuals relative to lean controls (76). Strong epidemiologic associations have emerged between high levels of circulating cytokines and cancer risk (77–79) and poor prognosis (80–83).

### Impact of Inflammation and Adipose Tissue Dysfunction on Intrinsic Metabolic Pathways

Obesity-associated adipose tissue dysfunction is driven by a portrait of signaling pathways that destabilize normal energy storage mechanisms and promote tumor growth. Leptin, in addition to its impact on satiety, also has wide-reaching metabolic effects on other body tissues. By signaling through its receptor (LEP-R), which is upregulated in certain cancers (84, 85), leptin can activate the PI3K/Akt/mTOR and Janus kinase/signal transducer and activator of transcription (JAK/STAT) pathways (86, 87). The former drastically reshapes internal metabolic pathways by the mechanisms discussed above. The latter also impacts several metabolic pathways important to cell growth and proliferation. For instance, binding of leptin to LEP-R activates JAK, which results in STAT3 phosphorylation and dimerization. Dimerized STAT3 translocates to the nucleus, where it acts as a transcription factor to upregulate many genes involved in glycolysis (88, 89), with evidence that this activity is HIF-1α-dependent (90). In fact, STAT3 interaction with HIF-1α is required to mount a full response to hypoxic conditions (91). Adiponectin, on the other hand, is counterregulatory to leptin signaling in many respects. Adiponectin activates AMPK downstream of its receptors (AdipoR1 and 2) in a variety of tissues to conserve energy by inhibiting anabolic processes (92). Numerous studies have demonstrated a robust antiproliferative effect of adiponectin. Indeed, intracellular activation of AMPK, in concert with an impressive array of additional mechanisms, mediate adiponectin’s strong growth-suppressive effects *in vitro* and *in vivo* (70, 93, 94).

Obesity-associated metabolic dysregulation also promotes the recruitment of immune cells to the adipose tissue. Expression of monocyte chemoattractant protein-1 is increased in the adipose tissue of obese subjects, prompting an influx of monocytes fated for differentiation into macrophages (95, 96). The resulting surge of cytokine secretion, particularly TNFα, is known to be a cause of obesity-associated insulin resistance in the adipose tissue by promoting serine phosphorylation of IR substrate-1, thereby blocking the propagation of downstream insulin signaling (97, 98). Cytokine-mediated insulin resistance in adipocytes results in compensatory hyperinsulinemia, which promotes tumorigenesis by the mechanisms enumerated above. Furthermore, insulin resistance in adipocytes permits unabated basal lipolysis (99). This results in release of free fatty acids (FFAs), perhaps providing additional substrate to cancer cells *via* uptake of exogenous FFAs (100). The saturated FFAs released by adipocytes also serve as ligands for toll-like receptor 4 (TLR4), which activate the nuclear factor-kappa B (NF-κB) signaling pathway in macrophages to perpetuate inflammatory activity and cytokine production (101, 102). Intriguingly, cancer cells themselves can exert a paracrine lipolytic effect on nearby adipocytes, in turn supplying tumors with energy and driving cell growth and metastasis (103–105).

**Figure 2:**
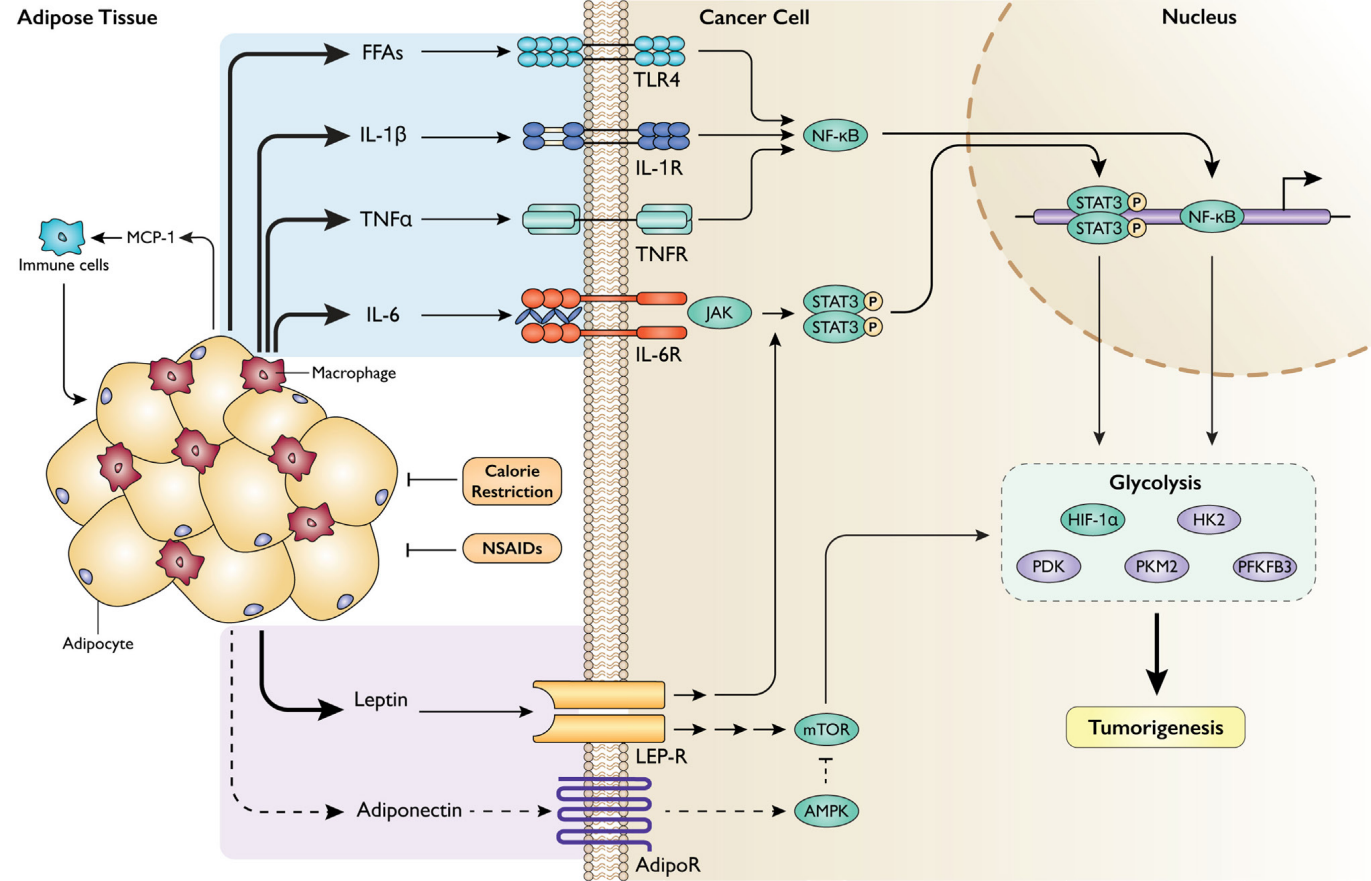
Impact of obesity-associated adipose tissue dysfunction and inflammation on cancer cell metabolism. Adipose tissue in the obese state becomes the site of profound inflammatory activity, recruiting immune cells that secrete a barrage of cytokines that can act on neighboring cancer cells to promote tumorigenesis through metabolic levers. MCP-1, monocyte chemoattractant protein-1; FFAs, free fatty acids; IL-1β, interleukin-1 beta; TNFα, tumor necrosis factor alpha; IL-6, interleukin-6; TLR4, toll-like receptor 4; (IL-1/TNF/IL-6)R, receptor; NF-κB, nuclear factor-kappa B; JAK, Janus kinase; STAT3, signal transducer and activator of transcription 3. Obesity also disrupts the endocrine functions of adipose tissue. LEP-R, leptin receptor; AdipoR, adiponectin receptor; NSAIDs, non-steroidal anti-inflammatory drugs. Intervention approaches are shown in orange.

Additionally, cytokines have numerous direct effects on cancer cells. IL-6 binds to IL-6 receptor and, similar to leptin, activates JAK/STAT3 signaling (106). Consistent with STAT3-mediated activation of glycolysis, administration of IL-6 has been shown to enhance glycolysis *in vitro* by STAT3-dependent upregulation of hexokinase 2 and PFKFB3 (107). IL-6, TNFα, IL-1β, and FFAs all activate NF-κB through signals downstream of binding to their respective receptors (TLR4 for FFAs) (101, 106, 108). NF-κB is a powerful mediator of inflammatory signaling by functioning as a transcription factor for dozens of target genes that have pleiotropic tumorigenic effects (109). NF-κB also restructures metabolic pathways to support cell growth, including activation of aerobic glycolysis (110) and angiogenesis (111); these effects are achieved at least in part by transcriptional and posttranslational activation of HIF-1α by the NF-κB pathway (112). Common to JAK/STAT3 and NF-κB signaling is the upregulated transcription of various cytokines, including IL-6 and TNFα. This has the effect of perpetuating proinflammatory signals as these cytokines can activate the pathways that promote their synthesis (113). Lastly, immune cells in the adipose tissue milieu give off an oxidative burst of reactive oxygen species to kill foreign or dangerous cells. It has been hypothesized that these radicals can contribute to DNA damage and mutagenesis in nearby tissues (114). This calls attention to the fact that obesity-associated inflammation in the adipose tissue can have multifaceted effects on tumorigenesis. Integration of these signaling pathways is displayed in Figure 2.

### Targeting the Metabolic Effects of Obesity on Adipose Tissue Dysfunction

Significant effort has been invested to translate an understanding of obesity-associated inflammation into actionable prevention and therapeutic strategies for cancer. Interestingly, several existing pharmacologic agents have proven useful to reduce cancer burden by targeting inflammatory pathways. Non-steroidal anti-inflammatory drugs (NSAIDs) are a class of pharmacologic agents that inhibit cyclooxygenase 1 and 2 (COX1 and 2). COX2 expression is inducible by inflammatory stimuli, and it synthesizes signaling lipids known as prostanoids from arachidonic acid (115). Prostanoids, particularly prostaglandin E2, promote tumorigenesis through diverse mechanisms reviewed extensively elsewhere (116). A series of studies published by Rothwell et al. show that daily use of aspirin, a NSAID, reduced cancer incidence (117), mortality (117, 118), and metastasis (119). In another study, NSAID use reduced breast cancer recurrence in overweight and obese women by 52% (120). Moreover, supplementation with celecoxib, a specific COX2 inhibitor, was shown to dramatically reduce the number of preneoplastic colorectal polyps in patients with familial adenomatous polyposis, a hereditary condition that portends a nearly 100 percent risk of colorectal cancer (121). Thus, there is strong evidence in support of prophylactic NSAID regimens to mitigate the impact of obesity-associated inflammation on cancer risk and progression.

Dietary approaches have also demonstrated efficacy to reduce obesity-associated inflammation. Several studies in humans report lower circulating levels of cytokines and C-reactive protein in overweight and obese individuals after undergoing significant (>10%) weight loss (122–126). However, the degree of weight loss seems critical to predict successful anti-inflammatory effects, as the impact of more modest levels of weight loss on inflammatory factors is less consistently observed. Preclinical studies using mouse models are accordingly conflicted on the benefits of weight loss relevant to inflammation and cancer, with those studies employing a robust CR regimen achieving more favorable reductions in inflammatory markers and tumor growth (127) than studies with only modest dietary weight loss strategies (128). Despite the efficacy of dietary and pharmacologic approaches directed against obesity-associated inflammation, certain pathologies might prevent these strategies from improving outcomes in obese individuals. Importantly, NF-κB is constitutively active in a wide variety of solid tumors and hematologic malignancies (109). Given that NSAID supplementation and weight loss regimens both improve obesity-associated inflammation in part by attenuating NF-κB activity downstream of inflammatory signals, these interventions might have limited use in patients with autonomous activation of this signaling pathway. Again, it is clear that case-specific genetic alterations should frame preventive and therapeutic strategies to dismantle the obesity–cancer connection.

## Nutrient Availability

### Mechanistic Connections between Nutrient Availability and Autophagy

Body tissues, including tumors, receive nutrients and oxygen from circulation in order to support cell maintenance and growth. However, incidental metabolic stressors, such as hypoxia, energy scarcity, or growth factor reduction, require cells to sustain these functions through alternative means of macronutrient acquisition. In response to metabolic stress, cells can induce autophagy, a conserved catabolic process in which cellular cargo such as protein aggregates and organelles are degraded into their component parts and recycled. Upon induction of autophagy *via* nutrient deprivation, intracellular cargo is enveloped in double-membraned autophagososomes, which then fuse with lysosomes where the contents are degraded and released into the cytoplasm, where they can be recycled to metabolic pathways. Autophagy induction can allow cells to survive in low nutrient environments until supplies are replenished (129).

**Figure 3:**
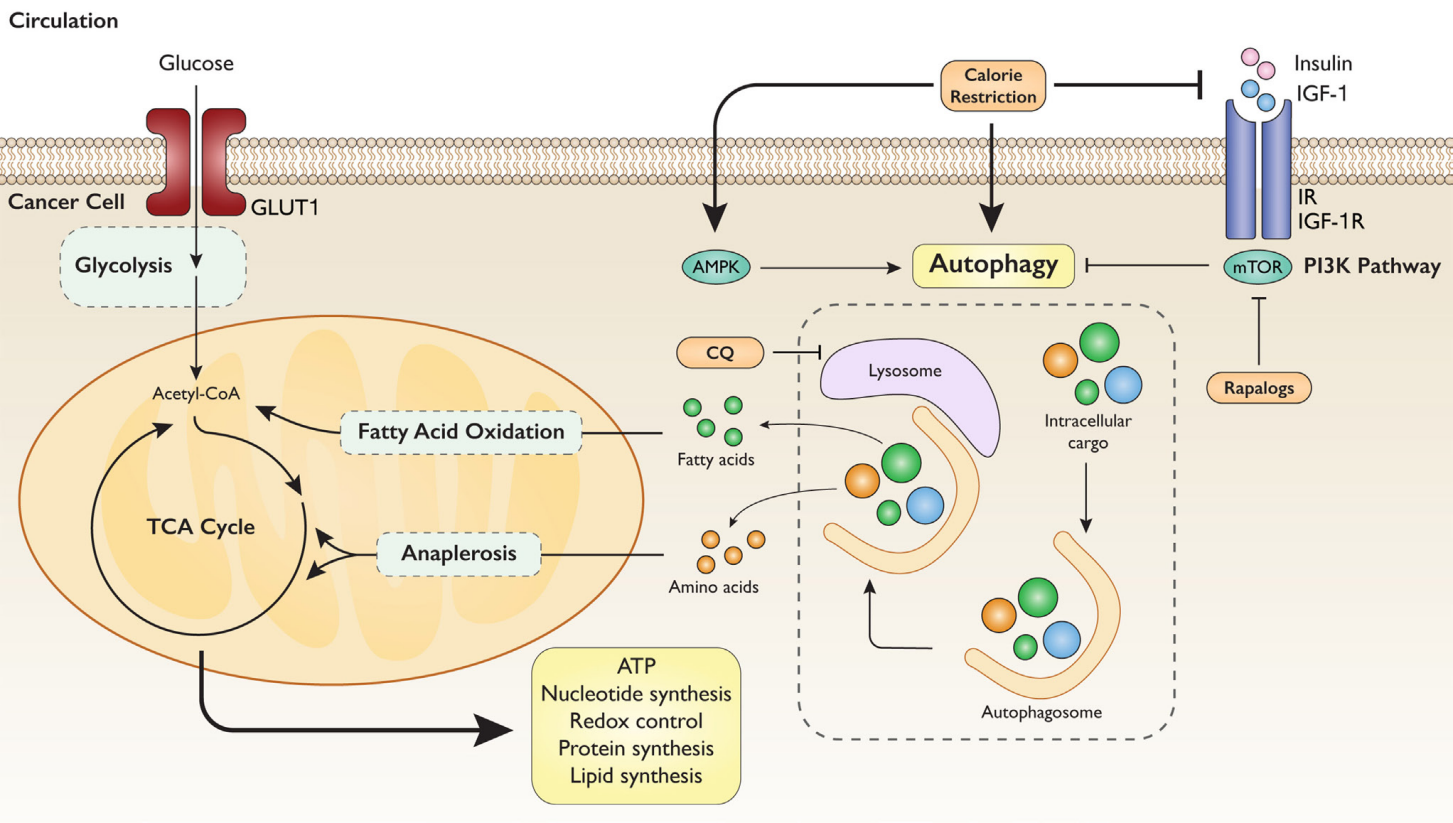
Autophagy mediates intrinsic metabolic response to extrinsic nutrient signals. Model illustrating the tumorigenic aspect of autophagy-mediated metabolic reprogramming. Nutrient deprivation signals the degradation of intracellular cargo to utilize constituent substrates for energy and biosynthesis. Calorie restriction strongly induces autophagy through several mechanisms. Simultaneous inhibition of autophagy with CQ (chloroquine) exerts sufficient metabolic stress to block tumorigenesis.

Tumors are prone to metabolic stress by virtue of outpacing the support of vasculature. In consequence, hypoxia and nutrient scarcity are frequently imposed on tumors, particularly those cell populations in the center of solid tumors. In malignant cells, the role of autophagy is complex and integrally linked to intrinsic cell metabolism. By recycling intracellular cargo, autophagy liberates amino acids, carbohydrates, and fatty acids to supply energetic and biosynthetic substrates for glycolysis, the TCA cycle, and fatty acid oxidation. This lends metabolic plasticity to cancer cells during changing environmental conditions (130). A summary of these metabolic interactions is displayed in Figure 3.

### Manipulating Nutrient Availability in Cancer Prevention and Therapy

Considering the survival advantage achieved by activating autophagy in malignant cells under metabolic stress, inhibiting autophagy in cancer has emerged as a rational chemotherapeutic paradigm. Indeed, several clinical trials are currently underway to determine the effect of autophagy inhibition, particularly by the lysosomal inhibitor chloroquine, in a number of different cancers in combination with chemotherapy or radiotherapy. Be that as it may, autophagy also confers several tumor suppressive effects, such as controlling oxidative stress and genomic instability (129). Therefore, efforts to undermine cancer metabolism by simultaneously restricting intrinsic and extrinsic substrate availability are being explored. Given the regulation of autophagy by nutrient availability, dietary regimens may also affect autophagy in cancer cells. CR, CR mimetics, and fasting as well as fasting mimicking diets have all been shown to induce autophagy in several tissues, including tumors (131–134). Work recently published by our group showed that a calorie restricted regimen combined with autophagy inhibition achieved more effective reduction in tumor growth than CR or autophagy inhibition alone (135). Furthermore, a growing body of evidence demonstrates that fasting can sensitize tumors to chemotherapy and radiotherapy (9). As such, the combined stress of dietary restriction and anticancer therapy may be sufficient to overwhelm a cancer cell.

In the context of overnutrition, autophagy deficiency appears to confer resistance to obesity and insulin resistance in mice fed a high fat diet (136, 137). In addition, autophagy has been shown to have a key role in obesity-associated cancers, such as pancreatic and liver cancer (138, 139). Certainly, obesity poses a unique array of obstacles relevant to targeting autophagy in cancer. The characteristic hyperglycemia accompanying the obese state reduces the likelihood of tumors encountering energetic scarcity, and the abundance of growth factors leads to sustained activation of the PI3K pathway, which is known to inhibit autophagy. Therefore, the metabolic excess of obesity removes the need of autophagy from an energetic or substrate availability perspective. However, the survival advantage conferred by an environment replete with nutrients and growth factors is well established. This draws attention to the need for further research on the role of autophagy mediating the impact of obesity on cancer metabolism.

## Conclusion

As summarized in Figures 1–3, this review considers lessons learned from obesity and cancer research to discuss promising extrinsic and cellular-intrinsic targets for cancer prevention, particularly for breaking the obesity–cancer link. Potential cell–extrinsic targets include systemic and locally produced hormones, growth factors, adipokines and other adipose-derived factors, and inflammatory mediators. Obesity-responsive host characteristics interact with several cancer cell–intrinsic factors, including alterations in components of the PI3K/Akt/mTOR pathway, and metabolic characteristics. Thus, given the complexity of the obesity–cancer relationship, and the expanding global epidemic of obesity, new precision medicine-based approaches that target extrinsic and cell–intrinsic interactions discussed in this review are urgently needed to break the obesity–cancer link.

## Author Contributions

SD, CO, and SH conducted the literature review and wrote the final manuscript. SD illustrated the figures.

## Conflict of Interest Statement

The authors declare that the research was conducted in the absence of any commercial or financial relationships that could be construed as a potential conflict of interest.
